# Generative Artificial Intelligence in Medical Education—Policies and Training at US Osteopathic Medical Schools: Descriptive Cross-Sectional Survey

**DOI:** 10.2196/58766

**Published:** 2025-02-11

**Authors:** Tsunagu Ichikawa, Elizabeth Olsen, Arathi Vinod, Noah Glenn, Karim Hanna, Gregg C Lund, Stacey Pierce-Talsma

**Affiliations:** 1 College of Osteopathic Medicine, University of New England, 11 Hills Beach Road, Biddeford, ME, 04005, United States, 1 2076022880; 2 College of Osteopathic Medicine, Rocky Vista University, Parker, CO, United States; 3 College of Osteopathic Medicine, Touro University California, Vallejo, CA, United States; 4 McCombs School of Business, University of Texas at Austin, Austin, TX, United States; 5 Morsani College of Medicine, University of South Florida, Tampa, FL, United States

**Keywords:** artificial intelligence, medical education, faculty development, policy, AI, training, United States, school, university, college, institution, osteopathic, osteopathy, curriculum, student, faculty, administrator, survey, cross-sectional

## Abstract

**Background:**

Interest has recently increased in generative artificial intelligence (GenAI), a subset of artificial intelligence that can create new content. Although the publicly available GenAI tools are not specifically trained in the medical domain, they have demonstrated proficiency in a wide range of medical assessments. The future integration of GenAI in medicine remains unknown. However, the rapid availability of GenAI with a chat interface and the potential risks and benefits are the focus of great interest. As with any significant medical advancement or change, medical schools must adapt their curricula to equip students with the skills necessary to become successful physicians. Furthermore, medical schools must ensure that faculty members have the skills to harness these new opportunities to increase their effectiveness as educators. How medical schools currently fulfill their responsibilities is unclear. Colleges of Osteopathic Medicine (COMs) in the United States currently train a significant proportion of the total number of medical students. These COMs are in academic settings ranging from large public research universities to small private institutions. Therefore, studying COMs will offer a representative sample of the current GenAI integration in medical education.

**Objective:**

This study aims to describe the policies and training regarding the specific aspect of GenAI in US COMs, targeting students, faculty, and administrators.

**Methods:**

Web-based surveys were sent to deans and Student Government Association (SGA) presidents of the main campuses of fully accredited US COMs. The dean survey included questions regarding current and planned policies and training related to GenAI for students, faculty, and administrators. The SGA president survey included only those questions related to current student policies and training.

**Results:**

Responses were received from 81% (26/32) of COMs surveyed. This included 47% (15/32) of the deans and 50% (16/32) of the SGA presidents (with 5 COMs represented by both the deans and the SGA presidents). Most COMs did not have a policy on the student use of GenAI, as reported by the dean (14/15, 93%) and the SGA president (14/16, 88%). Of the COMs with no policy, 79% (11/14) had no formal plans for policy development. Only 1 COM had training for students, which focused entirely on the ethics of using GenAI. Most COMs had no formal plans to provide mandatory (11/14, 79%) or elective (11/15, 73%) training. No COM had GenAI policies for faculty or administrators. Eighty percent had no formal plans for policy development. Furthermore, 33.3% (5/15) of COMs had faculty or administrator GenAI training. Except for examination question development, there was no training to increase faculty or administrator capabilities and efficiency or to decrease their workload.

**Conclusions:**

The survey revealed that most COMs lack GenAI policies and training for students, faculty, and administrators. The few institutions with policies or training were extremely limited in scope. Most institutions without current training or policies had no formal plans for development. The lack of current policies and training initiatives suggests inadequate preparedness for integrating GenAI into the medical school environment, therefore, relegating the responsibility for ethical guidance and training to the individual COM member.

## Introduction

Artificial intelligence (AI) is a technology capable of performing tasks traditionally requiring human intelligence [1]. AI has a long-standing presence in medicine across clinical, educational, and administrative domains [2-4]. Generative artificial intelligence (GenAI) technologies are a subset of AI that can create new content.

In the clinical domain, GenAI has demonstrated proficiency in performing tasks ranging from passing the United States Medical Licensing Examination to providing empathetic patient communication [5,6]. At a more advanced level, these tools have answered real-world medical questions with more factual accuracy and more empathy than human physicians [7,8]. Such capabilities highlight GenAI’s potential as a pivotal tool in both the learning environment of medical students and the broader context of patient care. However, the integration of GenAI into medical education raises important questions regarding the ethical, legal, and practical implications of its use.

Increased computing power, the development of a user-friendly conversational interface that lowers the technical barriers to use, and the availability to the public at little or no direct cost have made this technology nearly as available as web-based search engines or document spell-checking for medical educators and students. This has stimulated a great deal of interest by all constituencies in medicine and medical education. GenAI is only 1 component of the general field of AI. However, with the recent nearly ubiquitous availability to the general population in the United States, the yet clearly defined risks and benefits have significant implications for the short term in all aspects of medicine and the need for training and policies for medical trainees.

The rapid evolution of GenAI highlights the responsibility of medical schools to take a proactive approach to adapt their curricula and policies to harness the benefits of these technologies while mitigating potential risks. How medical schools currently fulfill their responsibilities is unclear. There are published reports highlighting individual AI-related training programs, as well as recommendations for AI curriculum, content, delivery, and challenges in medical schools [9-11]. While insightful, they do not describe the full educational landscape of US medical schools that grant either DO or MD degrees. This is particularly crucial in Colleges of Osteopathic Medicine (COMs) in the United States, which account for a significant and growing proportion of the country’s medical student population. Understanding the current landscape of GenAI policies and training in COMs is essential for identifying gaps, setting benchmarks, and guiding future initiatives aimed at effectively integrating GenAI into medical education.

GenAI has rapidly become nearly ubiquitous in the United States and has the potential for significant benefits and risks. It is unclear whether COMs have included training or policy guidance in this domain. This study aimed to describe the status of policy and training, specifically in one aspect of AI, GenAI, for medical students, faculty, and administrators, as well as near-term plans for policy and training development at COMs. This analysis will provide an overview of the current state of GenAI integration in osteopathic medical education, which will demonstrate opportunities for future development.

## Methods

### Study Design and Population

This descriptive cross-sectional study targeted US COMs that held full accreditation by the Commission on Osteopathic College Accreditation as of the end of the 2022‐2023 academic year. These COMs have at least 1 graduating class, ensuring that they possess a comprehensive experience with the full spectrum of undergraduate medical education. Approximately 28% of all US medical students are enrolled in COMs [12,13] in academic settings ranging from large public research universities to small private institutions. Therefore, we believe that studying COMs will offer a representative sample of the current GenAI integration in US medical education.

### Ethical Considerations

Before initiating contact with potential participants, the institutional review board (number 0723-10) of the University of New England, Biddeford, Maine, granted this project an exemption status. Participation in the study was voluntary, and informed consent was provided in both the email invitation and beginning of the survey. Data collection procedures were designed for privacy and confidentiality with deidentification of respondents. There was no compensation for survey participation.

### Survey Development and Data Collection

Due to the novel and rapidly developing field of GenAI, a survey was developed using an iterative process to obtain the availability, content, and development plans for training and policies for students, faculty, and administrators. The survey was designed to prioritize the general details of these domains. This strategy was to maximize the survey participation and to provide direction for potential future projects. The design was led by team members with experience in the user interface (GCL), survey development (GCL and SPT), COM medical curriculum development (GCL and SPT), and COM administrative management and operations (GCL and SPT). The survey was tested before implementation with a convenience sample of administrators and students to ensure that the questions were straightforward and the web-based survey system was usable. The order of survey items was the same for all participants in each group, with each question being presented on an individual screen. However, the surveys used an adaptive methodology to expose participants only to pertinent questions. For example, only those participants who answered that they currently provided training would be asked about the content of the training. If a COM stated that they do not have a GenAI policy, they would be asked about future development.

Data were collected using a survey distributed via a web-based tool (Qualtrics XM). The recruitment for participation was sent by an email directly to the potential participant. The recruitment email described the project purpose and survey details, including that the survey was on the web, anonymous, and no incentives were provided for their participation. No personal data were collected, including the respondent’s IP address. Two separate surveys were developed: one for the deans of the COMs and another for the presidents of the Student Government Association (SGA). The dean’s survey included questions about current and planned GenAI policies and training for students, faculty, and administrators, as well as questions about the content of existing policies and training (Multimedia Appendix 1). Recognizing that students are unlikely to have knowledge of policy, curriculum planning, or those related to faculty or administrators, the SGA president’s survey exclusively encompassed questions about current student policies and training (Multimedia Appendix 2). In both the dean and SGA president recruitment email, the recipient was informed that if there was a more appropriate survey responder, they may forward the email to that person, such as, the dean to an appropriate administrator, and the SGA president to a student.

Each dean and SGA president recruitment email included a unique survey URL to ensure that only 1 response represented each COM for each category. Qualtrics provides distribution data that are separate from the survey results. This allowed follow-up emails to nonresponders while maintaining the anonymity of the data. Data were collected from July 28, 2023, to September 14, 2023.

### Data Analysis

Descriptive statistics were used to analyze the survey results. Response rates for both surveys were calculated as the number of completed surveys as a percentage of total COMs surveyed. The number of started but not completed surveys was calculated as a percentage of total COMs surveyed. For each COM not providing training or having a policy, the status of development was reported as the percentage of COMs surveyed without that characteristic. Due to the anonymity of the respondents and the institutional overlap of the dean and SGA presidents, no statistical comparison between the 2 groups was made.

## Results

### Response Rates

Of the 32 COMs surveyed, 47% (15/32) deans and 50% (16/32) SGA presidents completed the survey. Five surveys overlapped deans and SGA presidents. The dean or SGA president responded from 81% (26/32) of the COMs surveyed, providing a comprehensive understanding of the COMs. All surveys started were completed (100%).

### GenAI Policies for Students

A vast majority of COMs reported a lack of established policies regarding the use of GenAI by students. Specifically, 93% (14/15) of deans and 88% (14/16) of SGA presidents indicated that their institutions had no student-focused GenAI policies. Among the few COMs with existing policies, the scope was primarily limited to GenAI use in graded assignments. Of the COMs with no policy, 79% (11/14) had no formal plans for policy development. The stages of planning for student policy are shown in Table 1.

**Table 1.** Status of student generative artificial intelligence policy and training development (Colleges of Osteopathic Medicine without policy or training).

	Student GenAI^a^ policy	Student mandatory education	Student elective education
Total surveys, n	14	14	15
Status, n (%)			
Not working on a policy or education	3 (21.4)	3 (21.4)	8 (53.3)
Informal conversations	8 (57.1)	8 (57.1)	3 (20)
Workgroup in place	1 (7.1)	3 (21.4)	2 (13.3)
Being drafted and under review	2 (14.3)	0 (0)	1 (6.7)
Approved to take effect after July 1, 2023	0 (0)	0 (0)	1 (6.7)

a GenAI: generative artificial intelligence.

### GenAI Training for Students

Only 1 COM was identified as having mandatory student training, which focused entirely on the ethics of using GenAI. None of the COMs offered any elective training. Most COMs had no formal plans to provide mandatory (11/14, 79%) or elective (11/15, 73%) training. The stages of planning for student training are shown in Table 1.

### GenAI Policies for Faculty or Administrators

None of the COMs studied had a GenAI policy for faculty or administrators. Similar to the students, 80% (12/15) had no formal plans to develop one. The stages of planning for faculty or administrator policy are shown in Table 2.

**Table 2.** Status of faculty or administrator generative artificial intelligence policy and training development for Colleges of Osteopathic Medicine (COMs) with no policy or training.

		Faculty/administrator policy	Faculty/administrator training
Total surveys, n		15	10
Status, n (%)			
	We are not working on a policy or training	6 (40)	2 (20)
	Informal conversations	6 (40)	3 (30)
	Workgroup in place	2 (13.3)	2 (20)
	Being drafted and under review	1 (6.7)	3 (30)
	Approved and will take effect after July 1, 2023	0 (0)	0 (0)

### GenAI Training for Faculty or Administrators

Only 33.3% (5/15) of COMs had initiated faculty or administrator-focused GenAI training. These predominantly covered basic use and ethical considerations. Except for examination question development, there was no specific focus on skills to enhance educational efficiency or reduce workload (Table 3). Fifty percent (5/10) of the COMs without faculty or administrator training had no formal plans to develop training (Table 2).

**Table 3.** Content of current faculty or administrator generative artificial intelligence training.

	Deans, n (%)
Total surveys	5 (100)
How to use the technology	4 (80)
Benefits/limitations of the technology	4 (80)
Ethics of using it	3 (60)
Legal perspective on using it	2 (40)
Development of examination questions	2 (40)

## Discussion

### Principal Findings

Our survey uncovers a pronounced gap in GenAI policies and training across US COMs, with the vast majority of institutions surveyed lacking formal policy guidelines (93% dean responses and 88% SGA president responses), and of the COMs with no current student policies, 79% (11/14) had no formal plans for future development. Furthermore, no COMs described any student GenAI elective training, with 73% (11/15) reporting no plans for mandatory educational programs. This underscores an urgent GenAI training imperative for medical schools to prepare future physicians for the imminent AI-enhanced health care landscape. Little has been done to support COM faculty to address these needs as no COMs surveyed had a formal policy regarding Gen AI for faculty or administration, 80% (12/15) did not have a plan to develop one, and only 33% (5/15) had focused training mainly in the realm of utilization and ethical considerations.

### Comparison With Prior Work

In a recent national survey of US postsecondary schools, 8% had GenAI policies in place [14]. In that report, the focus of the policies was not described. If these were related to students, it is comparable with the data of this project, where 7% (1/15) of the deans or 12% (2/16) of the SGA presidents responded that they had student GenAI policies. In our sample of student GenAI policies, the focus was on using GenAI in graded assignments. While there were few COMs with student-focused policies, none of the COMs had faculty or administrator policies.

The survey results indicated that the status of COM AI policies is unlikely to change significantly in the near future, with few COMs having formal plans to evaluate and develop GenAI policies. The 21% (3/14) of COMs with formal plans for student policies and 20% (3/15) with plans for faculty or administrator policies demonstrate that they are far less engaged than the postsecondary programs, in which 57% are evaluating and developing policies [14].

As with policy, training for COM students, faculty, and administrators is minimal and does not focus on enabling students, faculty, or administrators to increase productivity, improve effectiveness, or decrease workload. Because the majority do not have formal plans to develop training, this situation is unlikely to change significantly in the near future.

### Implications for Future Practice

The rapid advancement of AI technologies, including GenAI, necessitates a proactive stance from medical education institutions to integrate these tools effectively and ethically into teaching, learning, and clinical practice. COMs must move more quickly to develop AI policies and training. However, we do not propose indiscriminately replicating the nascent policies or training approaches of other institutions, which may not be appropriate for their institution. Furthermore, we caution against a hasty and thoughtless development process merely for the sake of establishing provisional measures. Instead, we propose that medical educators and administrators use the growing body of resources to strategically and methodically create policies and training resources using interdisciplinary teams and continually improve them as future GenAI innovations progressively transform the paradigm of technology-assisted human labor.

One example of resources to be reviewed is the study by Chan [15] that presented an AI policy framework integrating their local data and the UNESCO (United Nations Educational, Scientific and Cultural Organization) AI policy guidance [16]. This policy framework is divided into 3 dimensions, governance, operational, and pedagogical, and can also be used as a competency framework, as shown in Table 4.

**Table 4.** Artificial intelligence (AI) education policy framework [15].

Domain	Explanation	Content	Leadership
Pedagogical	Teaching and learning aspects of AI integration.	Rethinking assessments and examinations. Developing student holistic competencies/generic skills Preparing students for the AI-driven workplace Encouraging a balanced approach to AI adoption	Teachers
Operational	Practical implementation of AI in university settings	Monitoring and evaluating AI implementation Providing training and support for teachers, staff, and students in AI literacy	Teaching and learning and IT staff
Governance	Governance considerations surrounding AI usage in education	Understanding, identifying, and preventing academic misconduct and ethical dilemmas Addressing governance of AI: data privacy, transparency, accountability, and security Attributing AI technologies Ensuring equity in access to AI	Senior management

Further frameworks for describing AI literacy and learner competencies have emerged [9,10,17-20] and can form a starting point for COMs when developing a curriculum consistent with their institution’s educational mission and existing pedagogical architecture. Building upon this framework, in addition to work done internally, the growing body of published content resources can be accessed and, where appropriate, integrated into their development process. Some resources may be adapted from general educational domains, including skills such as writing [21] or faculty development of course content [22]. Other resources are specific to clinical care [20,23], education [24], or ethical use [25,26]. By adopting and evolving these frameworks with growing evidence-based resources, medical schools can ensure that their curricula not only cover the operational aspects of GenAI but also address the ethical, social, and professional implications.

This general framework is appropriate for learners at any developmental stage. However, as in other areas of medical education, the learners’ level of training [11,27] must be considered. For faculty or administrators, responsibilities in developing, integrating, and operationalizing the curriculum must also be considered [28].

In addition to the trainee level, medical school policy makers and educators must consider the systems in which future physicians will work. Physicians should be part of a team with diverse backgrounds and professional training to be most effective. With further AI development, these teams will include AI-powered computer assistants. The team must know how to interact effectively and appropriately with this new “team member,” including how it affects the patients and families they care for. This awareness is similar to the early assessments of the effects of electronic health records during clinical encounters [29,30].

Implementing GenAI competencies or any new content is a challenge with an already crowded curriculum. We propose that GenAI be integrated into the current system, where other tools are used to minimize the negative effect. When trainees learn to search and evaluate background scientific publications, GenAI can be incorporated where appropriate as one of the tools they are trained with. Furthermore, when practicing for clinical encounters, whether an actual clinical encounter or their objective structured clinical exams, using GenAI as a tutor may potentially reinforce their preparation. There are many similar uses that will integrate GenAI as a tool and not necessitate a significant increase in curriculum time and may additionally make other aspects of their curriculum more effective. However, these efforts will need further evaluation.

By developing clear policies and offering robust training, medical schools can ensure that future physicians are adept at leveraging GenAI to improve health care outcomes while navigating the ethical and professional complexities it presents.

### Limitations

This study’s findings must be interpreted in light of several limitations. The availability of data limits this project. Ongoing assessment is needed that includes a larger group of medical schools, including those that grant either doctor of osteopathic medicine or doctor of medicine degrees. In addition, other aspects of the physician’s life cycle (graduate medical education, clinical practice, and continuing education) must be studied.

The rapidly evolving nature of GenAI requires institutional policies and training initiatives that can quickly adapt, necessitating ongoing research to capture these developments accurately.

### Conclusions and Future Directions

Most COMs do not provide AI policy guidance or training for medical students, faculty, or administrators. There also does not seem to be an appropriate prioritization by COMs to remedy this deficiency. While many philosophers, including the great baseball legend Yogi Berra, have opined that “It is difficult to make predictions, especially about the future” [31], this difficulty does not negate medical schools’ responsibility while waiting for the future to become clear. They must assess future physicians’ needs and implement appropriate training and guidance in their programs. If the COMs do not lead, their trainees will be unprepared for the future. This risks inappropriate use of AI and the medical equivalent to the lawyer who used GenAI to submit a brief in court that included fabricated references or “hallucinations” [32].

Future research should explore effective strategies for implementing GenAI education and policy development, including interdisciplinary approaches and stakeholder engagement.

## Supplementary material

10.2196/58766 Multimedia Appendix 1 COM (College of Osteopathic Medicine) dean survey.

10.2196/58766 Multimedia Appendix 2 SGA (Student Government Association) president survey.

## Abbreviations

AI: artificial intelligence
COMs: Colleges of Osteopathic Medicine
GenAI: generative artificial intelligence
SGA: Student Government Association
UNESCO: United Nations Educational, Scientific and Cultural Organization
