# Supplementary material for: Generative Artificial Intelligence in Medical Education—Policies and Training at US Osteopathic Medical Schools: Descriptive Cross-Sectional Survey
Source: JMIR Med Educ. 2025 Feb 11;11:e58766. doi: 10.2196/58766 (PMC11835596; doi:10.2196/58766)
Supplement: Multimedia Appendix 1 [file mededu-v11-e58766-s001.docx]

Appendix A - COM Dean Survey

**Dean Survey**

Thank you for taking our survey. If you would like to read the Participant Information Sheet, please <link to Participant Information Sheet>to access it (it will open in a new tab). Otherwise, select the option below and click "Next page."

Q1 Does your COM currently (as of July 1, 2023) have any policies related to the STUDENT use of Generative Artificial Intelligence (GenAI) such as ChatGPT?

- Yes
- No

If Q1=Yes, the respondent was taken to Q2

If Q1=No, the respondent was taken to Q3

Q2 Which areas do the policies cover? Check all that apply.

- Submitted student assignments

- Individual learning activities (e.g. individual studying time, searching/summarizing information, making study guides/flashcards/notes, etc.)

- Graded examinations

- Written communication (e.g., emails, letters of support, promotional content, etc.)

- Clinical simulations

- Individual/team patient notes during clinical rotations

- Unsure/Prefer not to answer

- Other: Please specify.__________________________________________

Q3 At this moment in time, which of the following best describes the state of your COM developing a policy regarding STUDENT use of GenAI?

- We are not working on a policy.

- We have had informal conversations about a policy but have taken no formal action.

- We have a workgroup in place to evaluate need and make recommendations.

- We have a policy that is being drafted and/or under review.

- We have a policy that is approved to take effect after July 1, 2023.

- Unsure/Prefer not to say

Q4 Did your COM have GenAI included in the FORMAL MANDATORY STUDENT curriculum this last school year (2022-2023)?

- Yes

- No

If Q4 = Yes, the respondent was taken to Q5

If Q4 = No, the respondent was taken to Q6

Q5 What areas were covered? Check all that apply.

- How the technology works

- Benefits/Limitations of the technology

- Ethics of using it

- Legal perspective on using it

- Prompt engineering/How to ask GenAI questions

- Interprofessional communications

- Patient communication or education

- Language translation

- Student self-education (summary of journal articles, developing flashcards, etc.)

- Clinical care (differential diagnoses, reformatting case presentations by setting, etc.)

- Unsure/Prefer not to say

- Other: Please specify.__________________________________________

Q6 At this moment in time, which of the following best describes the state of your COM's plans to include GenAI in your FORMAL MANDATORY STUDENT CURRICULUM?

- We have no plans to do so.

- We have had informal conversations but have not taken any formal action.

- We have a workgroup in place to evaluate the need and make recommendations.

- We have curriculum drafted and/or under review.

- We have approved curriculum that will take effect after July 1, 2023.

- Unsure/Prefer not to say

Q7 Did your COM have GenAI included in the ELECTIVE STUDENT curriculum or OPTIONAL TRACK this last school year (2022-2023)?

- Yes

- No

If Q7 = Yes, the respondent was taken to Q8

If Q7 = No, the respondent was taken to Q9

Q8 What areas were covered? Check all that apply.

- How the technology works

- Benefits/Limitations of the technology

- Ethics of using it

- Legal perspective on using it

- Prompt engineering/How to ask GenAI questions

- Interprofessional communications

- Patient communication or education

- Language translation

- Student self-education (summary of journal articles, developing flashcards, etc.)

- Clinical care (differential diagnoses, reformatting case presentations by setting, etc.)

- Unsure/Prefer not to say

- Other: Please specify.__________________________________________

Q9 At this moment in time, which of the following best describes the state of your COM's plans to include GenAI in your ELECTIVE STUDENT curriculum or OPTIONAL TRACK?

- We have no plans to do so

- We have had informal conversations but have not taken any formal action.

- We have a workgroup in place to evaluate the need and make recommendations.

- We have curriculum drafted and/or under review.

- We have approved curriculum that will take effect after July 1, 2023.

- Unsure/Prefer not to say

Q10 Does your COM currently (as of July 1, 2023) have any policies related to the FACULTY/ADMINISTRATION use of Generative Artificial Intelligence such as ChatGPT?

- Yes

- No

If Q10 = Yes, the respondent was taken to Q11

If Q10 = No, the respondent was taken to Q12

Q11 What areas do the policies cover?

- Grading examinations or assignments

- Developing examination questions

- Creating curricular learning content (PowerPoint slides, scenarios, handouts, cases, etc.)

- Written communication (e.g., emails, letters of support, promotional content, etc.)

- Policy development

- Student candidate assessment (e.g., for admissions, scholarships, etc.)

- Faculty candidate assessment (e.g., for hiring, tenure, promotion, etc.)

- Rubric development

- Simulation/OSCE (development or assessment)

- Unsure/Prefer not to say

- Other: Please specify.__________________________________________

Q12 At this moment in time, which of the following best describes the state of your COM developing a GenAI policy for FACULTY/ADMINISTRATION?

- We are not working on a policy.

- We have had informal conversations about a policy but have taken no formal action.

- We have a workgroup in place to evaluate need and make recommendations.

- We have a policy that is being drafted and/or under review.

- We have a policy that is approved to take effect after July 1, 2023.

- Unsure/Prefer not to say

Q13 Did your COM have FACULTY/STAFF development/training on utilizing GenAI during the last school year (2022-2023)?

- Yes

- No

If Q13 = Yes, the respondent was taken to Q14

If Q13 = No, the respondent was taken to Q15

Q14 What areas were covered?

- How to use the technology

- Benefits/limitations of the technology

- Ethics of using it

- Legal perspective on using it

- Prompt engineering/How to ask GenAI questions

- Interprofessional communications

- Patient communication or education

- Language translation

- Student self-directed learning aids (summary of journal articles, development of flashcards, study guides, etc.)

- Clinical care (differential diagnoses, reformatting case presentations by setting, etc.)

- Development of learning aids or artifacts (PowerPoint slides, handouts, etc.)

- Assessment of student assignments

- Development of exam questions

- Evaluation of curriculum

- Unsure/Prefer not to say

- Other: Please specify.__________________________________________

Q15 At this moment in time, which of the following best describes your COM's plans to offer FACULTY/STAFF development/training?

- We have no plans to do so.

- We have had informal conversations about doing so but have taken no formal action.

- We have a workgroup in place to evaluate the need and make recommendations.

- We have plans under review/consideration.

- We will offer FACULTY/STAFF development/training on using GenAI after July 1, 2023.

- Unsure/Prefer not to say
