# Supplementary material for: Generative Artificial Intelligence in Medical Education—Policies and Training at US Osteopathic Medical Schools: Descriptive Cross-Sectional Survey
Source: JMIR Med Educ. 2025 Feb 11;11:e58766. doi: 10.2196/58766 (PMC11835596; doi:10.2196/58766)
Supplement: Multimedia Appendix 2 [file mededu-v11-e58766-s002.docx]

Appendix B - SGA President Survey

**SGA President Survey**

Thank you for taking our survey. If you would like to read the Participant Information Sheet, please click <<link to the Participant Information Sheet>> to access it (it will open in a new tab). Otherwise, click "Next page."

Q1 Does your COM currently (as of July 1, 2023) have any policies related to the STUDENT use of Generative Artificial Intelligence (GenAI) such as ChatGPT?

- Yes

- No

If Q1=Yes, the respondent was taken to Q2

If Q1=No, the respondent was taken to Q3

Q2 Which areas do the policies cover? Check all that apply.

- Submitted student assignments

- Individual learning activities (e.g. individual studying time, searching/summarizing information, making study guides/flashcards/notes, etc.)

- Graded examinations

- Written communication (e.g., emails, letters of support, promotional content, etc.)

- Clinical simulations

- Individual/team patient notes during clinical rotations

- Unsure/Prefer not to answer

- Other Please specify. _________________________________

Q3 Did your COM have GenAI included in the FORMAL MANDATORY STUDENT curriculum this last school year (2022-2023)?

- Yes

- No

If Q3=Yes, the respondent was taken to Q4

If Q3=No, the respondent was taken to Q5

Q4 What areas were covered? Check all that apply.

- How the technology works

- Benefits/Limitations of the technology

- Ethics of using it

- Legal perspective on using it

- Prompt engineering/How to ask GenAI questions

- Interprofessional communications

- Patient communication or education

- Language translation

- Student self-education (summary of journal articles, developing flashcards, etc.)

- Clinical care (differential diagnoses, reformatting case presentations by setting, etc.)

- Unsure/Prefer not to say

- Other Please specify. _________________________________

Q5 Did your COM have GenAI included in the ELECTIVE STUDENT curriculum or OPTIONAL TRACK this last school year (2022-2023)?

- Yes

- No

If Q5=Yes, the respondent was taken to Q6

If Q5=No, the respondent was taken to the end

Q6 What areas were covered? Check all that apply.

- How the technology works

- Benefits/Limitations of the technology

- Ethics of using it

- Legal perspective on using it

- Prompt engineering/How to ask GenAI questions

- Interprofessional communications

- Patient communication or education

- Language translation

- Student self-education (summary of journal articles, developing flashcards, etc.)

- Clinical care (differential diagnoses, reformatting case presentations by setting, etc.)

- Unsure/Prefer not to say

- Other: Please specify. _________________________________
